# Supplementary material for: Identification of Freezing-Responsive microRNAs and Their Targets in Chinese Jujube by Small RNA and Degradome Sequencing
Source: Int J Mol Sci. 2026 Sep 4;27(17):7912. doi: 10.3390/ijms27177912 (PMC13566890; doi:10.3390/ijms27177912)
Supplement: Supplementary file 1 [file ijms-27-07912-s001.zip › Supplementary Material S2/novel_miR_156.pdf]

Provisional ID : novel_miR_156
Score total : 0.4
Score for star read(s) : -1.3
Score for read counts : 0
Score for mfe : 0.1
Score for randfold : 1.6
Score for cons. seed :
Total read count : 296
Mature read count : 296
Loop read count : 0
Star read count : 0

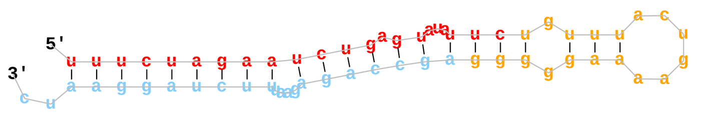

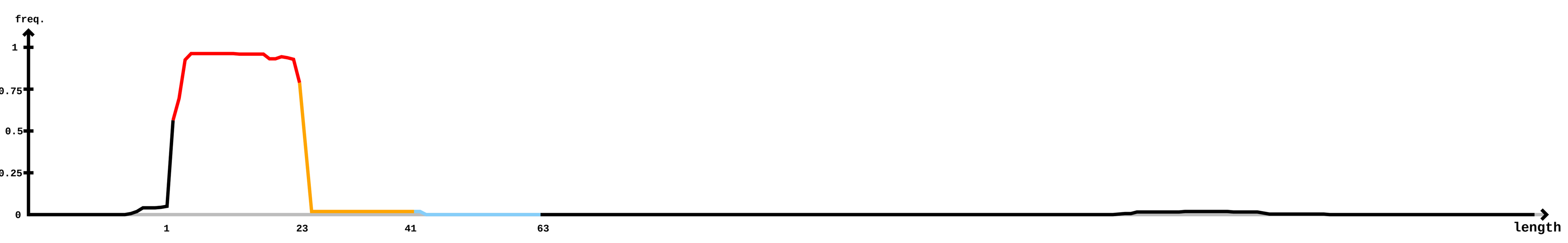

Mature Star

```
5'- aaggcugauuccauuaaagagauuucuagaaucugaguauauucuguuuacugaaaagggggagccagagaauuucuaggaaucuauuucucauaaagacaaagaaaaaugauugcaaagaaugcaagaugagugggaaucuuuggagaaaaaucauaauuaugaguugguuaagcuuccaacaggaaagaaggcacuaaagaaaaaauggguauucaagcucaagaaacaaagcccgauuggugguuaa  -3'   exp
    .(((((..((((......(((((((((((((((.((...((((.(((.....))).))))))))))....))))))))))))).((((((.((((((............(((((.....)))))...........)))))))))))..((((....))))(((((........))))).))))....))).)).........(((((......)))))........(((((.....).))))..   reads   mm   sample
    ...............aaagagauuucuagaauU.........................................................................................................................................................................................................................   1   1   LT-1
    ...............aaagagauuucuagaaucugagu....................................................................................................................................................................................................................   1   0   LT-1
    ................aagagauuucuagaaucugagu....................................................................................................................................................................................................................   2   0   LT-1
    ................aagagauuucuagaaucugagu....................................................................................................................................................................................................................   1   0   JCK-3
    ................aagagauuucCagaaucugaguau..................................................................................................................................................................................................................   1   1   LT-1
    .................agagauuucuagaaucugagu....................................................................................................................................................................................................................   2   0   LT-2
    .................agagauuucuagaaucugagu....................................................................................................................................................................................................................   1   0   LT-3
    .................agagauuucCagaaucugagu....................................................................................................................................................................................................................   1   1   LT-1
    .................agagauuucCagaaucugaguau..................................................................................................................................................................................................................   1   1   JCK-1
    .................agagauuucuagaaucugaguaua.................................................................................................................................................................................................................   1   0   JCK-2
    .................agagauuucuagaaucugaguaua.................................................................................................................................................................................................................   1   0   JCK-1
    ....................gauuucCagaaucugagu....................................................................................................................................................................................................................   1   1   JCK-1
    .....................auuucuagaaucugaguauauu...............................................................................................................................................................................................................   1   0   JCK-1
    .....................auuucuagaaucugaguauauucu.............................................................................................................................................................................................................   1   0   LT-1
    ......................uuucuagaaucugaguauau................................................................................................................................................................................................................   2   0   JCK-3
    ......................uuucuagaaucugaguauUu................................................................................................................................................................................................................   1   1   LT-1
    ......................uuucuagaaucugaguauauu...............................................................................................................................................................................................................   1   0   JCK-1
    ......................uuucuagaaucugaguauUuu...............................................................................................................................................................................................................   2   1   JCK-2
    ......................uuucuagaaucugaguauauC...............................................................................................................................................................................................................   1   1   JCK-2
    ......................uuucuagaaucugaguauUuu...............................................................................................................................................................................................................   3   1   JCK-3
    ......................uuucuagaaucugaguauauu...............................................................................................................................................................................................................   1   0   JCK-3
    ......................uuucuagaaucugaguauauC...............................................................................................................................................................................................................   2   1   LT-1
    ......................uuucuagaaucugaguauauu...............................................................................................................................................................................................................   1   0   LT-3
    ......................uuucuagaaucugaguauauu...............................................................................................................................................................................................................   1   0   JCK-2
    ......................uuucuagaaucugaguauUuu...............................................................................................................................................................................................................   5   1   LCK-3
    ......................uuucuagaaucugaguauUuu...............................................................................................................................................................................................................   1   1   JCK-1
    ......................uuucCagaaucugaguauauu...............................................................................................................................................................................................................   2   1   LT-3
    ......................uuucCagaaucugaguauauuc..............................................................................................................................................................................................................   10   1   JCK-3
    ......................uuucuagaaucugaguauauuc..............................................................................................................................................................................................................   9   0   JCK-1
    ......................uuucCagaaucugaguauauuc..............................................................................................................................................................................................................   12   1   JCK-1
    ......................uuucCagaaucugaguauauuc..............................................................................................................................................................................................................   12   1   JCK-2
    ......................uuucuagaaucugaguauauuc..............................................................................................................................................................................................................   9   0   LT-2
    ......................uuucuagaaucugaguauauuc..............................................................................................................................................................................................................   22   0   LCK-2
    ......................uuucuagaaucugaguauauuc..............................................................................................................................................................................................................   17   0   LCK-1
```

```
                      Mature                                                        Star
aaggcugauuccauuaaagagauuucuagaaucugaguauauucuguuuacugaaaagggggagccagagaauuucuaggaaucuauuucucauaaagacaaagaaaaaugauugcaaagaaugcaagaugagugggaaucuuuggagaaaaaucauaauuaugaguugguuaagcuuccaacaggaaagaaggcacuaaagaaaaaauggguauucaagcucaagaaacaaagcccgauugguggguuaa
......................uuucuagaaucugaguauauuc..............................................................................................................................................................................................................   1  0  LT-3
......................uuucuagaaucugaguauauuc..............................................................................................................................................................................................................  12  0  LCK-3
......................uuucuagaaucugaguauauuc..............................................................................................................................................................................................................  16  0  JCK-2
......................uuucCagaaucugaguauauuc..............................................................................................................................................................................................................   2  1  LT-2
......................uuucuagaaucugaNuauauuc..............................................................................................................................................................................................................   1  1  LCK-1
......................uuucuagaaucugaguauauuc..............................................................................................................................................................................................................   9  0  JCK-3
......................uuucuagaaucugaguauauuc..............................................................................................................................................................................................................   7  0  LT-1
......................uuucCagaaucugaguauauuc..............................................................................................................................................................................................................   3  1  LT-1
.......................uucuagaaucugaguauauC...............................................................................................................................................................................................................   2  1  LT-3
.......................uucCagaaucugaguauauuc..............................................................................................................................................................................................................   4  1  LT-2
.......................uucCagaaucugaguauauuc..............................................................................................................................................................................................................   7  1  JCK-3
.......................uucCagaaucugaguauauuc..............................................................................................................................................................................................................   2  1  LT-3
.......................uucuagaaucugaguauauuc..............................................................................................................................................................................................................   6  0  JCK-1
.......................uucCagaaucugaguauauuc..............................................................................................................................................................................................................   3  1  JCK-1
.......................uucuagaaucugaguauauuc..............................................................................................................................................................................................................   2  0  JCK-3
.......................uucuagaaucugaguauauuc..............................................................................................................................................................................................................   5  0  JCK-2
.......................uucuagaaucugaguauauuc..............................................................................................................................................................................................................   1  0  LT-3
.......................uucuagaaucugaguauauuc..............................................................................................................................................................................................................   3  0  LT-1
.......................uucCagaaucugaguauauuc..............................................................................................................................................................................................................   2  1  JCK-2
.......................uucCagaaucugaguauauuc..............................................................................................................................................................................................................   2  1  LT-1
.......................uucuagaaucugaguauauucu.............................................................................................................................................................................................................   1  0  LT-3
.......................uucuagaaucugaguauauucu.............................................................................................................................................................................................................   2  0  JCK-2
........................ucuagaaucugaguauauu...............................................................................................................................................................................................................   5  0  JCK-1
........................ucuagaaucugaguauauu...............................................................................................................................................................................................................   1  0  LT-1
........................ucuagaaucugaguauauu...............................................................................................................................................................................................................   1  0  LT-2
........................ucuagaaucugaguauauC...............................................................................................................................................................................................................   2  1  JCK-2
........................ucuagaaucugaguauUuu...............................................................................................................................................................................................................   2  1  LT-1
........................ucuagaaucugaguauauu...............................................................................................................................................................................................................   3  0  JCK-2
........................ucuagaaucugaguauauu...............................................................................................................................................................................................................   5  0  JCK-3
........................ucuagaaucugaguauauuc..............................................................................................................................................................................................................   4  0  JCK-2
........................ucuagaaucugaguauauuc..............................................................................................................................................................................................................   7  0  JCK-1
........................ucuagaaucugaguauauuc..............................................................................................................................................................................................................   3  0  LT-3
........................ucuagaaucugaguauauuc..............................................................................................................................................................................................................   7  0  LT-1
........................ucuagaaucugaguauauuc..............................................................................................................................................................................................................  14  0  JT-1
........................ucuagaaucugaguauauuc..............................................................................................................................................................................................................   3  0  LCK-3
........................ucuagaaucugaguauauuc..............................................................................................................................................................................................................   3  0  JCK-3
........................ucuagaaucugaguauauuc..............................................................................................................................................................................................................   9  0  LT-2
........................ucuagaaucugaguCuauuc..............................................................................................................................................................................................................   1  1  LT-2
........................ucCagaaucugaguauauuc..............................................................................................................................................................................................................   2  1  LT-1
........................ucuagaaucugaguauauuA..............................................................................................................................................................................................................   2  1  JCK-2
.........................cuagaaucugaguauauu...............................................................................................................................................................................................................   2  0  LT-2
.........................cCagaaucugaguauauu...............................................................................................................................................................................................................   1  1  LT-1
.........................cuagaaucugaguauauuc..............................................................................................................................................................................................................   1  0  JCK-1
.........................cuagaaucugaguauauuc..............................................................................................................................................................................................................   3  0  LCK-3
.........................cuagaaucugaguauauuc..............................................................................................................................................................................................................   3  0  LT-2
.........................cuagaaucugaguauauuc..............................................................................................................................................................................................................   1  0  LT-1
.........................cCagaaucugaguauauucu.............................................................................................................................................................................................................   1  1  LT-1
........................................auucuguuuacugaaGagggggag..........................................................................................................................................................................................   3  1  JCK-2
........................................auucuguuuacugaaGagggggag..........................................................................................................................................................................................   3  1  JCK-1
...................................................................................................................................................................................caacaggaaagaaggcacuaaaga...............................................   1  0  JCK-1
....................................................................................................................................................................................aacaggCaagaaggcacu....................................................   1  1  LT-3
......................................................................................................................................................................................caggaaagaaggcacuaaaga...............................................   1  0  JCK-2
......................................................................................................................................................................................caggaaagaaggcacuaaagaa..............................................   1  0  JCK-3
......................................................................................................................................................................................caggaaagaaggcacuaaagaa..............................................   1  0  JCK-2
..............................................................................................................................................................................................aaggcacuaaagaaUaaaugggua....................................   1  1  LT-3
```
